# The Association of Health and Income in the Elderly: Experience from a Southern State of Brazil

**DOI:** 10.1371/journal.pone.0073930

**Published:** 2013-09-13

**Authors:** Gerda G. Fillenbaum, Sergio L. Blay, Carl F. Pieper, Katherine E. King, Sergio B. Andreoli, Fábio L. Gastal

**Affiliations:** 1 Center for the Study of Aging and Human Development, Duke University Medical Center, Durham, North Carolina, United States of America; 2 Geriatric Research, Education and Clinical Center, Veterans Administration Medical Center, Durham, North Carolina, United States of America; 3 Department of Psychiatry, Federal University of São Paulo, Brazil (Escola Paulista de Medicina - UNIFESP), São Paulo, Brazil; 4 Department of Biostatistics and Bioinformatics, Duke University Medical Center, Durham, North Carolina, United States of America; 5 Department of Sociology, Duke University, Durham, North Carolina, United States of America; 6 Project Scientific Committee, Medical Director, Sistema de Saúde Mãe de Deus, Porto Alegre, Rio Grande do Sul, Brazil; Iran University of Medical Sciences, Iran (Islamic Republic Of)

## Abstract

**Objectives:**

In high income, developed countries, health status tends to improve as income increases, but primarily through the 50^th^-66^th^ percentile of income. It is unclear whether the same limitation holds in middle income countries, and for both general assessments of health and specific conditions.

**Methods:**

Data were obtained from Brazil, a middle income country. In-person interviews with a representative sample of community residents age ≥60 (N=6963), in the southern state of Rio Grande do Sul, obtained information on demographic characteristics including household income and number of persons supported, general health status (self-rated health, functional status), depression, and seven physician-diagnosed, self-reported health conditions. Analyses used household income (adjusted for number supported and economies of scale) together with higher order income terms, and controlled for demographics and comorbidities, to ascertain nonlinearity between income and general and specific health measures.

**Results:**

In fully controlled analyses income was associated with general measures of health (linearly with self-rated health, nonlinearly with functional status). For specific health measures there was a consistent linear association with depression, pulmonary disorders, renal disorders, and sensory impairment. For musculoskeletal, cardiovascular (negative association), and gastrointestinal disorders this association no longer held when comorbidities were controlled. There was no association with diabetes.

**Conclusion:**

Contrary to findings in high income countries, the association of household-size-adjusted income with health was generally linear, sometimes negative, and sometimes absent when comorbidities were controlled.

## Introduction

It has long been recognized that better health tends to be associated with higher socioeconomic status (SES), regardless of how SES is assessed (as social class, occupational category, educational accomplishment, property and ownership of goods, wealth), of how health is measured (in general terms: self-rated health, functional status; or by specific conditions, e.g., cardiovascular disorders, diabetes) [1-9], in countries that are rich [10], and in countries that are poor [11].

Implicit in this finding is the expectation that with increased income, health status will be better. But this has not always been borne out. Rather, studies have shown that the relationship between income and health is strongest at the lower income levels, generally tapering off markedly above the median on income, i.e., it is curvilinear [8,9,12]. While data from the nationally representative (U.S.) Health and Retirement Study suggest a linear relationship between household income and self-rated health, closer evaluation identifies a nonlinear association, with the effect decaying between the first (lowest) income tercile and the second, and again between the second and the third terciles for middle aged participants aged 51-61 [8]. Study of functional status in the same sample indicated that income had an effect only through the bottom two terciles [3]. A comparable situation held in the companion AHEAD study of persons 70 years of age and older. The Americans’ Changing Lives study of noninstitutionalized persons age 25 and over reported a “rapidly diminishing differences in health above the $20,000 level” (income as of 1986) [14], while report on persons age 18 and over from the 2007 National Health Interview Survey indicates that differences associated with income may vary by health condition [15].

Such studies appear to have been carried out primarily in high income countries, in particular the U.S. It has not always been possible to replicate these findings in middle income countries. In Brazil, data on persons age 60 and over from the 1998 and 2003 Brazilian National Household Surveys indicated that the association between income and functional status tapered off but was still present at the highest income levels [16]. On the other hand, the 9,000 strong Costa Rican Study of Longevity and Healthy Aging in adults age 60 and over found that older people at lower social status have, for certain measures, been found to have better health and health habits than those of higher social status [17].

It is unclear whether we should expect in a middle income country the association between income and health that is found in high income countries. A recent study [18], compared the health of representative samples of residents age 50 and over in the U.K. and Brazil by income terciles, and showed that while in both countries better income was associated with better functional status, those at the lowest income level in the UK had a better functional status than those at the highest income level in Brazil. This suggests that, while relative income has a similar impact on health, additional factors determine actual health status, and the stronger association of low income with health in high income countries may not apply in middle or low income countries.

The current study proposes to look at this issue, using information for Brazil, a fast-emerging middle income country (as defined by the World Bank), and for older persons there. While it is clear that, in Brazil, there is an association between income and health status (often measured by mobility or depression) [16,18-25], it is not clear whether the effect of income on health tapers off at around the median on income, as has been found in U.S. studies [8,9,12,14,15] or whether health is similarly affected throughout the income range. In addition, it is unclear whether income affects all health conditions similarly, since the non-linear association between income and health has typically examined self-rated health and functional status as outcomes. We propose to expand inquiry by looking at both general measures of health, and specific health conditions, since associations may differ. A key contribution is to avoid imposing functional form on income by using income as a continuous, rather than a categorical measure, and to take into account not only other demographic characteristics, but also other health conditions (which have rarely been controlled for).

## Methods

### Sample

The sample consisted of non-institutionalized community residents 60 years of age and older, selected by multistage, random sampling of nine homogeneous areas covering the state of Rio Grande do Sul, Brazil. Only one appropriate-age person per household was selected (selected at random if more than one age-eligible person was present). This southernmost state has a largely agro-industrial economy, and is populated primarily by descendants of European immigrants. Details on methods have been reported previously [26,27]. Data were gathered in September, 1995 from 7920 residents of urban and rural counties by trained, monitored interviewers, using structured face-to-face household surveys. Data entry problems were identified in one region, which was then excluded from analysis. In the remaining eight regions, 7040 residents were approached: 880 in each area. No proxy information was collected. Only 77 persons (1.1%) did not take part in the assessment, primarily refusals, yielding an overall response rate of 99% (N = 6963). The study was approved by the Ethics Committee of the Federal University of São Paulo. Participants gave oral consent. All data used in the current study were de-identified. For access to these data please contact the authors.

### Data available

The *primary independent variable* is household income recorded as multiples of the monthly minimum wage (MW) (US$83 in 1995 [28]), adjusted for number of people supported (including outside the immediate household). We took economies of scale into account by dividing total household income by number of persons supported raised to a power of 0.38. This was based on guidelines suggesting that so doing “best adjusts for differences in consumption needs across families of different sizes” [29,30]. We truncated the resulting value at 10MW since the tail of the income distribution was long and sparsely populated (with 1.1% of the sample). The resulting measure was used as a continuous variable. Whereas economic studies in Brazil use per capita information (i.e., income divided by the number of people supported), this frustrates comparison with the U.S., where procedures typically take into account economies of scale. Our approach better permits comparison. Family income was missing for 16.4% of the sample. To take this group into account a variable indicating whether household income was present or absent was included in analysis.

The *main dependent variables* were two general measures of health (each a continuous variable), and eight specific health conditions or classes of conditions (each dichotomized to indicate presence or absence). The general measures were self-rated health (0 = excellent, 1 = good, 2 = fair, 3 = poor, 4 = very poor), and functional status. The functional status measure is a 5-item unidimensional scale of instrumental and basic activities of daily living, specifically household activities (cleaning/home maintenance/meal preparation), taking medications, personal hygiene (bathing/combing hair/dressing/cutting nails), feeding self, and mobility (sitting down/getting up/lying down, walking, going up stairs) [31]. Each item was scored dichotomously (need help vs. able to perform independently). The number of items with which help was needed was summed (range 0-5).

The eight specific health conditions were: depression (ascertained by a validated Brazilian version of the Short Psychiatric Evaluation Schedule [32]), and self-report of medical care in the previous six months for diabetes, pulmonary disorders (bronchitis, pneumonia), renal disorders including urinary tract infections, sensory impairment (poor vision, poor hearing), musculoskeletal disorders (backache, osteoporosis, rheumatism/arthritis), cardiovascular disorders (heart disease, stroke, hypertension, varicosities), and gastrointestinal disorders. These summarize the array of health conditions inquired into, extend previous inquiry, and permit examination of possible alternate associations with income.

The *covariates* selected are those shown in previous studies to be associated with health status or health conditions. They include sex, age (continuous), race (White, Afro-Brazilian, other), and education (<4 years vs. 4+ years). In addition, *all health conditions* other than the health condition at issue were included as covariates in the analytic models, to permit examination of the condition at issue net of comorbidities.

### Statistical analyses

Descriptive statistics (mean, standard deviation, percentages, Wilcoxon) statistics were used to characterize the data. Multivariable OLS regression analyses were run for the continuous dependent variables (self-rated health, functional status), and multivariable logistic regression for dichotomously scored dependent variables. For each condition, 3 nested models were examined: income only; income plus demographics; income plus demographics plus health conditions other than the health condition at issue. To ascertain the shape of the association between income and the condition at issue, and to facilitate uniform examination across all dependent variables, each model was fit with terms for linear income, and all polynomials up to the fourth order. Prior analysis had indicated that no income term with a yet higher power was needed to fit the data. To determine whether higher order powers were needed to explain the association between income and the selected outcome, we assessed whether (a) there was an overall effect of income, tested by an omnibus impact of income for the four polynomials, and if significant, (b) whether there was a non-linear component to the relationship, tested with an omnibus test of the non-linear components (income^2^, income^3^, income^4^), controlling for the linear impact of income. If neither (a) nor (b) was significant, then it was assumed that income was not associated with the health condition at issue. If the higher order terms were significant, the relationship between income and the outcome variable was non-linear, and most easily seen after being graphed. If the higher order terms (income^2^, income^3^, income^4^) were not significant, but the overall test (a) was significant, then income was defined as a linear association with the outcome variable. Analyses were carried out using SAS version 9.2.

## Results

The sample (Table 1), is predominantly female, and white. The mean age is 70, two thirds have less than 4 years of education. A quarter live in households with adjusted incomes of less than 2MW, and over half in households with less than 3MW. Household size averages nearly three people. On average respondents consider their health to be fair, 39% could not perform all activities of daily living (ADL) independently. The presence of health problems ranged from 11% for diabetes to 63% for cardiovascular disorders. Those for whom household income information was missing (16.4%), were more likely to be older, female, and living in larger households. While they reported more ADL problems, they did not differ on self-rated health, and the only health condition on which they differed was a lower prevalence of renal disorders.

**Table 1:** Characteristics of the Analysis Sample (N = 6741).

		Household income adjusted by number of people supported^.38^		
	Total sample	Income report present (N=5818, 83.6%)	Income report absent (N=1145, 16.4%)	P-value^a^
	Mean (sd) or %	Mean (sd) or %	Mean (sd) or %	
Demographic characteristics				
Age (years)	70.2 (7.4)	69.9 (7.3)	71.7 (7.3)	<.0001
Sex				
Male	34.0	35.4	26.7	<.0001
Female	66.0	64.6	73.3	
Education				
<4 years	66.2	66.0	67.3	0.3927
≥4 years	33.8	34.0	32.7	
Race				
White	84.2	84.4	83.4	0.4665
Afro-Brazilian	6.8	6.6	7.9	
Other	9.0	9.0	8.7	
Health problems				
Depression	39.1	39.0	39.4	0.8222
Diabetes	11.0	10.8	11.8	0.3470
Pulmonary disorders	29.6	29.7	28.9	0.6008
Renal disorders	24.4	24.9	21.9	0.0327
Sensory impairment	77.7	77.9	76.7	0.3721
Musculoskeletal disorders	61.0	61.4	59.1	0.1526
Cardiovascular disorders	63.1	63.1	62.8	0.8463
Gastrointestinal disorder	18.3	18.7	16.3	0.0637
Self-rated health	1.75 (0.98)	1.75 (0.98)	1.78 (0.97)	0.5210 Cochran-Mantel-Haenszel 4 d.f., 7.38, P=.1169
Excellent (0)	9.5	9.8	8.0	
Good (1)	27.0	26.6	28.9	
Fair (2)	49.8	50.1	48.2	
Poor (3)	6.2	6.1	7.0	
Very poor (4)	7.6	7.5	8.0	
Functional status	0.97 (1.08)	0.64 (1.05)	0.83 (1.22)	<.0001
0 ADL^b^ impairments	60.8	62.0	54.4	
1 ADL impairment	24.5	23.9	27.9	
2+ ADL impairments	14.7	14.1	17.8	
Household size, number supported, income variables				
# living on household income	2.86 (1.58)	2.77 (1.53)	3.38 (1.76)	<.0001
Household income^c^ (MW)	4.61 (2.79)	4.61 (2.78)	2.00 (3.50)	<.0001
Household income (MW) adjusted for number supported^d^				
<1 MW	3.26 (1.94)	1.7% (96)		
1-<2 MW		24.1% (1400)		
2-<3 MW		30.7% (1786)		
3-<4 MW		16.6% (964)		
4-<5 MW		7.2% (419)		
5-<6 MW		8.3% (481)		
6-<7 MW		5.5% (321)		
7-<8 MW		4.6% (265)		
8-<9 MW		0.2% (12)		
9-<10 MW		0.2% (10)		
≥ 10 MW		1.1% (64)		

a Determined by Wilcoxon test

b ADL = activities of daily living (functional status scale ranges from 0-5 impairments)

c Family income coded in multiples of the minimum wage (MW) range: 0-10 (truncated at 10). Of the 1145 for whom no adjusted income is available, 16 reported family income but did not report the number of people supported.

d Income was adjusted for number of persons supported by dividing household income by number supported to the power of 0.38 (to take into account economies of scale).

Percentage may not total 100 because of rounding

**Figure 1:**
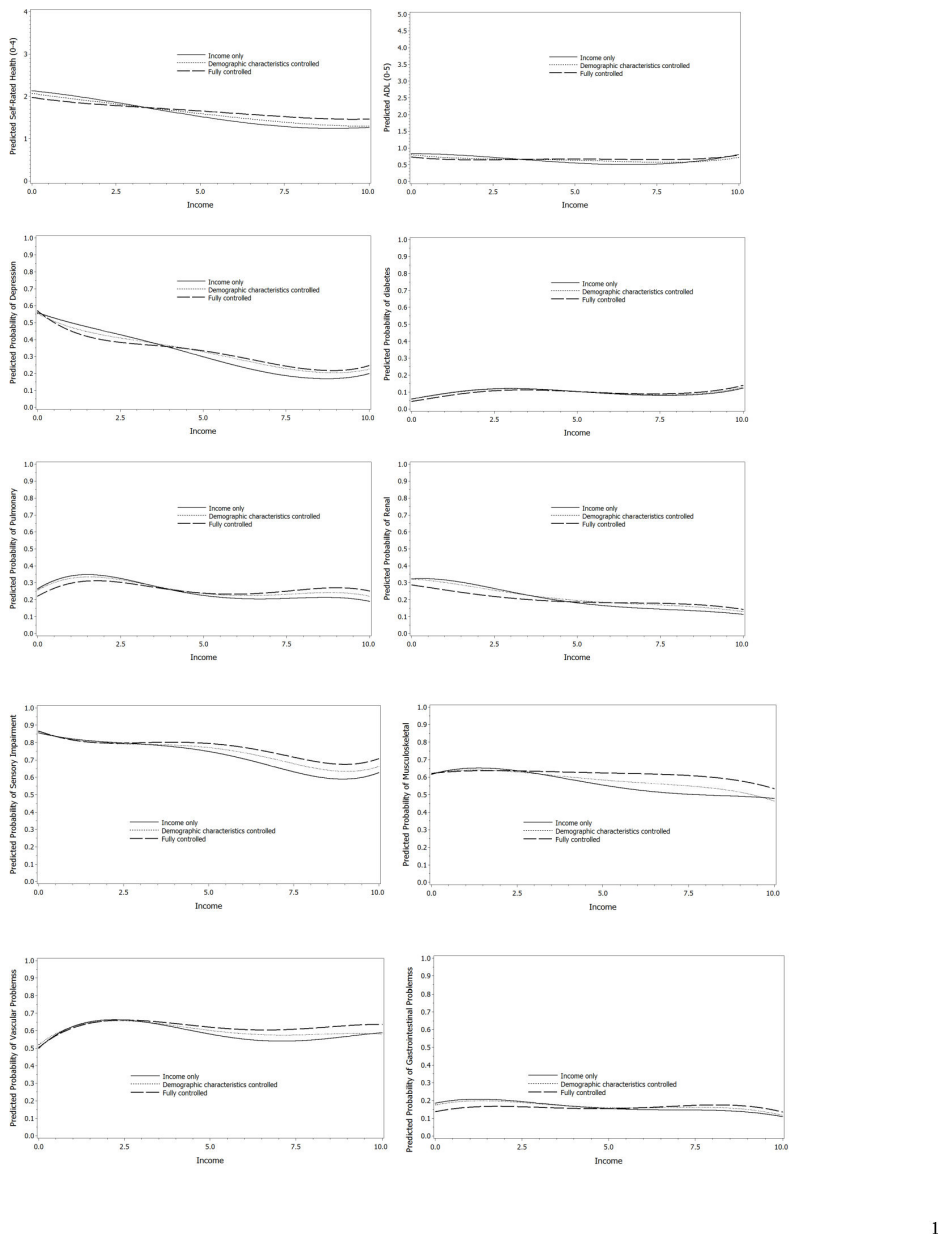
Health conditions (depression, diabetes, pulmonary disorders, renal disorders, sensory impairment, musculoskeletal disorders, cardiovascular disorders, gastrointestinal disorders) and health status (self-rated health, functional status), plotted against household income measured as a multiple of the minimum wage (MW) adjusted for number supported and for economies of scale. Lines show results controlling for income only, income + demographic characteristics, and income, demographic characteristics and health conditions other than the condition under consideration. For specific health conditions values on the vertical axis indicate predicted proportion with the disorder; for self-rated health 0 = excellent health, 4 = very poor health; for functional status the scale indicates predicted number of impairments in activities of daily living (possible range 0-5). The value on the horizontal axis is household income adjusted for number supported.

Table *2* provides summary information on the association between income and health conditions under three models (income only, controlled for demographic characteristics, and further controlled for other health conditions). The shapes of the associations are given in Figure 1.

**Table 2:** Summary findings based on household-size-adjusted income (multiple of minimum wage (MW)).

	Income only	Income + Demographics^a^	Income + Demographics + Health^b^
	OLS multivariable regression		
	F (p)	F (p)	F (p)
Self-rated health			
^c^ INC^1,2,3,4^	68.8 (<.0001)	34.0 (<.0001)	16.8 (<.0001)
^d^ INC^2,3,4^	1.3 (.2631)	0.2 (.9291)	0.6 (.6752)
Functional status			
INC ^1,2,3,4^	16.4 (.0001)	3.7 (.0026)	2.3 (.0417)
INC ^2,3,4^	9.1 (<.0001)	2.5 (.0405)	2.9 (.0212)
	Logistic regression		
		Wald χ^2^ (p)	Wald χ^2^ (p)
Depression			
INC ^1,2,3,4^	193.4 (<.0001)	86.4 (<.0001)	48.7 (<.0001)
INC ^2,3,4^	4.2 (.3756)	1.9 (0.7523)	3.92 (.4170)
Diabetes			
INC ^1,2,3,4^	10.3 (.0671)	8.4 (.1368)	7.5 (.1866)
INC ^2,3,4^	8.8 (.0639)	8.1 (.0892)	6.6 (.1616)
Pulmonary disorders			
INC ^1,2,3,4^	77.9 (.0001)	39.8 (<.0001)	19.8 (.0014)
INC ^2,3,4^	9.0 (.0612)	7.1 (.1296)	7.4 (.1147)
Renal disorders			
INC ^1,2,3,4^	99.4 (<.0001)	51.6 (<.0001)	20.5 (.0010)
INC ^2,3,4^	5.0 (.2876)	5.3 (.2537)	4.0 (.4053)
Sensory Impairment			
INC ^1,2,3,4^	84.2 (<.0001)	38.3 (<.0001)	20.6 (.0010)
INC ^2,3,4^	5.2 (.2649)	6.2 (.1831)	6.9 (.1409)
Musculoskeletal disorders			
INC ^1,2,3,4^	62.4 (<.0001)	26.8 (<.0001)	4.8 (.4450)
INC ^2,3,4^	4.9 (.3015)	6.9 (.1438)	2.5 (.6450)
Cardiovascular disorders			
INC ^1,2,3,4^	44.1 (<.0001)	19.4 (.0016)	6.8 (.2333)
INC ^2,3,4^	15.3 (.0042)	9.3 (.0534)	6.5 (.1671)
Gastrointestinal disorders			
INC ^1,2,3,4^	24.2 (.0002)	12.0 (.0346)	2.9 (.7163)
INC ^2,3,4^	4.5 (.3424)	4.5 (.3385)	2.8 (.5929)

Results of OLS regression (for self-rated health and activities of daily living), and logistic regression (for depression, diabetes, pulmonary disorders, renal disorders, sensory impairment, musculoskeletal disorders, cardiovascular disorders, gastrointestinal disorders) examining whether association between income and the dependent variable is linear or nonlinear (a) considering income only, (b) adjusted for demographic conditions, (c) further adjusted for health conditions.

a Demographic conditions controlled: age, sex, education (<4 years vs. ≥4 years), race (White, Afro-Brazilian, other)

b Health conditions controlled : depression, diabetes, pulmonary disorders, renal disorders, sensory impairment, musculoskeletal disorders, cardiovascular disorders, gastrointestinal disorders (except when the health condition is the dependent variable), each dichotomized to indicate presence vs. absence

c INC^1,2,3,4^ = F or Wald χ^2^ value for all income variables combined (income, income^2^, income^3^, income^4^, income present vs. absent)

d INC^2,3,4^ = F or Wald χ^2^ value for all higher order income variables (income^2^, income^3^, income^4^)

Under linear hypothesis testing, if the set of higher order income variables remains significant when all income variables are considered, a complex association exists between income and the outcome variable. If the set of higher order income variables does not reach statistical significance, the association of income with the outcome variable is indicated by the significance value of the entire set of income variables (INC^1,2,3,4^ + income present vs. absent), and is linear if this set of variables is significant.

### General measures of health

Both self-rated health and functional status (measured as number of ADL impairments) were associated with income—linear for self-rated health, non-linear for functional status, even when controlled for demographic characteristics and comorbidities. In uncontrolled analysis (i.e., income only (Model 1)), the predicted self-rated health of the poorest is just below “fair”, while at the highest income level it is closer to “good”, a difference of nearly a unit on the scale of excellent, good, fair, poor, very poor. With successive addition of demographic characteristics and comorbidities, the difference in self-rated health is nearly halved, ranging from “fair” for the lowest income, to midway between “fair” and “good” at the highest income.

The association of income with functional status (ADL performance) is more complex. Even with all covariates included, there is a nonlinear association of income with problems performing ADL activities. However, while statistically there is a strong association when only income variables are examined (Model 1), the association is severely attenuated in fully controlled analyses, with P-values reduced from P <.0001 to P <.02. Examination of the graph indicates that the predicted number of ADL impairments is ~0.8 for both lowest and highest adjusted household income regardless of model, with the lowest predicted number of ADL impairments (~0.65) at ~6.5MW.

### Specific health conditions

Specific health conditions differed in their association with income. Diabetes was never associated with income. Cardiovascular disorders had a nonlinear association with income when only income was in the model; this association became linear with the addition of demographic characteristics, and was no longer associated with income with the further inclusion of comorbidities. The remaining six health conditions (depression, pulmonary disorders, renal disorders, sensory impairment, musculoskeletal and gastrointestinal disorders), had a linear association with income (Model 1), and also when demographic characteristics were controlled (Model 2), but with the addition of comorbidities, musculoskeletal and gastrointestinal conditions, and cardiovascular disorders were no longer significantly associated with income, while depression, pulmonary and, renal disorders, and sensory impairment continued to have a linear association with income.

The probability of depression declines from a probability of ~0.55 at the lowest income to ~0.3 at the highest in the fully adjusted model. The predicted probability of pulmonary disorders *increases* from ~0.225 to ~0.29, while the probability of renal disorders declines from ~0.3 to nearly 0.2, and that of sensory impairment declines from ~0.85 to ~0.75.

Considering only those health conditions for which there is no longer an association with income when comorbidities are controlled, there is a maximum decline in the probability of musculoskeletal disorders of ~0.16 for the income only and demographic characteristics controlled models; for gastrointestinal disorders the probability is a decline of ~0.05. A more complex association is found for cardiovascular disorders. Under all models the probability of cardiovascular disorders *increases* from 0.5 to ~0.65 at 2.5MW, before declining (when income only is considered, but levelling off at 2.5MW when demographic characteristics are controlled, and further levelling and becoming nonsignificant with comorbidities controlled.

## Discussion

Although it is clear that in upper income countries, and in most middle income countries, there is an association between socioeconomic status and health, what is not clear is whether they share the same association between health and income *per se*. In our analysis of data from representative, elderly community residents of Rio Grande do Sul, a southern state in Brazil, we found that there was general agreement that persons with lower income had poorer health status than those with higher income, but we did not find the differential association of health with income found in upper income countries (steeper improvement in health with improvement in income at the lower levels). We looked at both general and specific measures of health, and unlike most studies, examined alternative measures within each category.

Different measures within the same category yielded different findings. General measures of health were represented by self-rated health, and functional status. Self-rated health consistently had a linear relationship with income, even after both demographic characteristics and comorbidities were controlled. Functional status consistently had a curvilinear relationship, although examination of the graph suggested that the association had little practical significance.

Among the specific measures (health conditions) three alternative associations with income were found, some with subtypes

1: No association between income and the health condition (diabetes)

2: A statistically significant association even in fully controlled models (i.e., including demographic characteristics, comorbidities). This variant had two subtypes–

2a. Consistent improvement in health throughout the income range (depression, sensory impairment)

2b. Deterioration in health as very low income improves, and then improvement in health with further increase in income (pulmonary problems)

3. A statistically significant association with income when demographic characteristics are controlled, but not when comorbidities are controlled. This variant also has two subtypes>:

3a. Consistent improvement in health throughout the income range (musculoskeletal and gastrointestinal disorders)

3b. Deterioration in health as very low income improves, and then improvement in health with further increase in income (cardiovascular disorders).

Finally, we should mention that this does not exhaust the possibilities, for Rosero-Bixby and Dow [17] found that in Costa Rica, for some conditions, lower social status people had better health than upper status people (metabolic syndrome, hypertension).

### Specific considerations

#### General measures of health.

Self-rated health, an indicator of current overall health status, and predictor of ill health, health service use, and death [23,25,35], maintained a linear association with income in all models. This finding is confirmed by data from the World Health Survey of ~5,000 people age 18 and over in Brazil [25], in which with age, education, and employment status controlled, economic level (measured by household assets) predicted self-rated health in groups with and without chronic disease or disability. Similarly, aggregated information from the Survey on Health and Wellbeing of Elders (SABE), of more than 10,000 community residents age 60 and over in seven cities in Latin America (Buenos Aires, São Paulo, Santiago, Havana, Mexico City, Montevideo) and the Caribbean (Bridgetown), in which age, gender, and country were controlled, also found that higher income predicted better self-rated health [23], as did findings in Costa Rica [17].

Functional status is a general measure that summarizes the impact of the health conditions present on level of independence [31,33]. We found a weak nonlinear association, with no consistent improvement. A graph of the findings was unspectacular. Comparison with other studies in the same geographic area is difficult, since they used more restricted measures of functional status. One study of older participants in the 1998 and 2003 Brazilian National Household Surveys [16,34],, found that improvement in self-reported ability to walk 100m continued at very high income levels, although the rate of improvement declined. In the SABE study mentioned above, income inequality was associated particularly with self-rated health, and with basic and instrumental activities of daily living [23]. Neither the 100m walk nor the SABE study controlled for health conditions.

#### Specific health conditions.

No association with income was found for diabetes. Absence of an association between income and diabetes is in agreement with findings for the aggregated SABE cities [23], but not the site-specific findings, where the prevalence of diabetes was significantly higher at the lowest educational level [36]. In another study, in Buenos Aires, sample members (age 18 and over) with low education had four times, and those with middle education had twice the adjusted odds of a diagnosis of diabetes than persons with a high level of education [37]. Note that the site-specific SABE study, and the Buenos Aires study used education and not income as a measure of socioeconomic status, and all sample members were city dwellers. Careful evaluation by House and colleagues has shown that both income and education are important determinants of health, with education predicting onset of a condition, and income predicting progression [2,38,39]. A recent systematic review [40] on the association of socioeconomic status (measured separately as level of education, occupation, or income), with incident diabetes, indicated little consistency across studies between improved socioeconomic status and incident diabetes. All but three studies, however, came from developed countries, two were from middle income countries (one country was Brazil [41], where an association between income and incident diabetes was found), and one was a low income country. Diabetes may be sensitive to the socioeconomic status measure used, but there may be alternative explanations, including a stronger genetic component than for some of the other health conditions examined, lack of recognition of the disease, and absence of seeking medical treatment for it within the past 6 months. Additional information which might have clarified this situation, information such as area or duration of current residence; diet, level of obesity, or medications used; lifetime occupation; frequency of seeking medical attention or difficulty accessing it, was not available.

Cardiovascular disorders were the only conditions where, in any model, there was a curvilinear association with income, but in so doing went contrary to expectation that with improvement in income there would be a consistent improvement in health (data from Costa Rica also showed lower cardiovascular risk factors in persons with lower socioeconomic status [17]). The reason for this is unclear, but may reflect the possible adverse impact of increased income on smoking, diet, exercise, selective survivorship, or under-diagnosis among persons with lower income. With the inclusion of comorbidities, there was no longer an association with income.

For all other specific health conditions except diabetes, there was a linear association with income, which was maintained when demographic characteristics were included. As with cardiovascular disorders, and likely for the same reasons, with initial increase in income, probability of pulmonary disorders increased, before declining. For other disorders (depression, renal disorders, sensory impairment, musculoskeletal and gastrointestinal disorders), increased income was salutary. After statistical control for comorbidities, however, a linear association with income remained only for depression, pulmonary and renal conditions, and sensory impairment. For musculoskeletal, cardiovascular disorders, and gastrointestinal disorders no statistically significant association with income remained.

Our data indicate that multiple patterns of association between income and health can exist. Increased income may not necessarily have a desirable effect, personal health behavior, and environmental circumstances (access to care), also play a role. The finding that income loses relevance for some conditions when comorbidities are present suggests that improvement in income alone is not adequate to reduce the probability of certain conditions, and that attention needs to be paid to health more broadly, in particular when treatment for certain conditions (e.g., cardiovascular disease, musculoskeletal disorders) may aggravate other conditions (e.g., gastrointestinal disorders).

In The U.S., all of the conditions examined have been shown to be associated with income, and for most conditions are attenuated above the 50^th^ or 66^th^ percentile of income [9,13,15]. Similarly, self-reported health, arthritis, and cardiovascular disorders have been shown to be associated with social class among men age 63-82 in Britain, with a less clear association for pulmonary disorders, diabetes, and musculoskeletal disorders [7,42].

In examining the association between income and health we have considered a broader array of health conditions than is usually the case, including summary assessments of health (self-rated health, functional status), as well as specific conditions, some of which are easier to identify and treat (e.g., sensory impairments), than are others. *Whilemany* studies categorize income, permitting only rough estimates of income/health associations, our measure of income is continuous. We take into account the number of people supported, adjusted for economies of scale (which has not always been done, and is not the usual procedure for studies for Brazil [28]). To determine whether the association between income and health is or is not linear, we included higher order polynomials of income in analysis, a strategy which permitted identification of the relationship present without imposing structure, and allowed uniform examination across health conditions. Graphs allow us to see the form of the association. To better understand the associations present we ran three increasingly controlled models for each health measure: income alone, with demographic characteristics controlled, and, not conventional in these studies, with comorbidities controlled.

We are aware of limitations in the current study. We have information only on current state. Prior work and income history, and childhood experience are relevant [43,44], but were unavailable. Self-rated health may not be consistent across socioeconomic status [45,46]. Health conditions were self-reported, but self-report has been found to be reasonably accurate, in particular for “obvious” or serious conditions [47]. Report only of conditions treated in the previous six months may have led to under-reporting, particularly for lower income people who may have had greater problems accessing health care. Household income information was missing for 16.4%. Those for whom household income was missing tended to be older, frailer, no longer married, women, living in larger households, but their health conditions were comparable to those providing income information. They may have influenced functional status findings, but are unlikely to have influenced findings for other health conditions. Our sample is age 60 and over, when health and income differences may be blunted [1,48,49]. The data are 15 years old, however nationally representative data from 1998 and 2003 show little change in health, functional status, or health service use as a function of income; inequalities persist [21]. Finally, our information is representative only of a southern section of Brazil. In other geographical areas of the country, particularly in the north where the income level is lower, health conditions, and the association between income and health may be different.

In summary, our findings add to those currently available for middle income countries [18-23,41]. Unlike many previous studies, we have looked at a broad array of health conditions, and we have controlled not only for demographic characteristics, but also for comorbidities. We typically found a linear association with income, even with demographic characteristics, including education, controlled. For cardiovascular and pulmonary disorders the association at the lowest income levels (but not at higher levels) was not benign. *A* *nonlinear* association with income was rarely found, for functional status it had little clinical meaning, and for cardiovascular disorders disappeared when comorbidities were controlled. While the association with income was maintained for certain conditions when comorbidities were controlled, for others it was lost, warning of the possible interplay among health conditions. Improvement in income, even at the lower income range, must be handled with care because of potential adverse effects. Health conditions may not be considered in isolation since, particularly in older persons, comorbidities may be present.
